# Supplementary material for: Basidiomycetous Yeast, Glaciozyma antarctica, Forming Frost-Columnar Colonies on Frozen Medium
Source: Microorganisms. 2021 Aug 7;9(8):1679. doi: 10.3390/microorganisms9081679 (PMC8398550; doi:10.3390/microorganisms9081679)
Supplement: Supplementary file 1 [file microorganisms-09-01679-s001.zip › microorganisms-1321078-supplementary/Supplemental files/Figure S1.pdf]

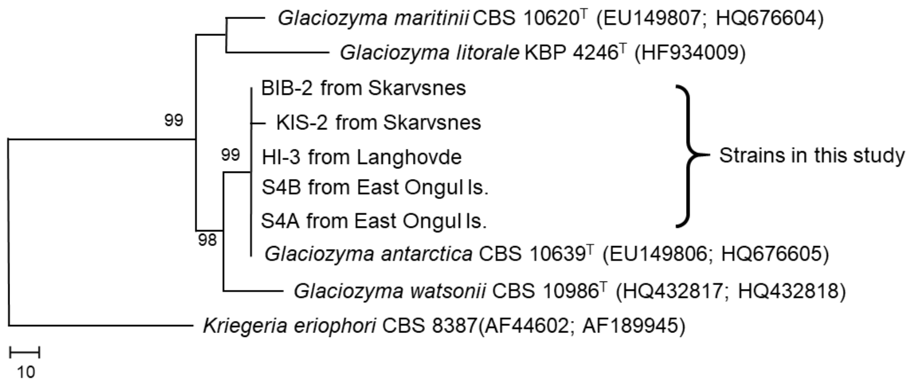

Figure S1. Maximum parsimony tree based combined ITS region and D1/D2 domains of the LSU rDNA sequences for strains from frost-columnar colonies and other species of *Glaciozyma*. The numbers given on branches given on branch are frequencies (>50%) with which a given branch appeared in 1,000 bootstrap replications. The scale indicates the numbers of nucleotide substitutions.
