# Supplementary material for: Basidiomycetous Yeast, Glaciozyma antarctica, Forming Frost-Columnar Colonies on Frozen Medium
Source: Microorganisms. 2021 Aug 7;9(8):1679. doi: 10.3390/microorganisms9081679 (PMC8398550; doi:10.3390/microorganisms9081679)
Supplement: Supplementary file 1 [file microorganisms-09-01679-s001.zip › microorganisms-1321078-supplementary/Supplemental files/Supplementary-r1.pdf]

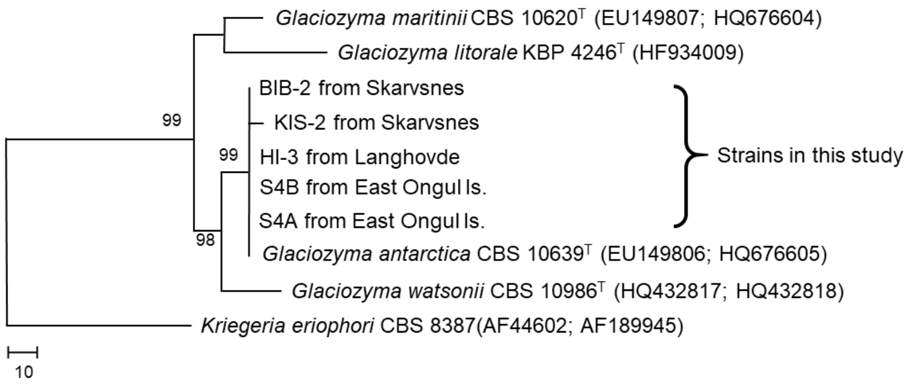

Figure S1. Maximum parsimony tree based combined ITS region and D1/D2 domains of the LSU rDNA sequences for strains from frost-columnar colonies and other species of *Glaciozyma*. The numbers given on branches given on branch are frequencies (>50%) with which a given branch appeared in 1,000 bootstrap replications. The scale indicates the numbers of nucleotide substitutions.

Table S1. Sample type to use in this study.

Locality	No. Samples examined	Sample type*				Sampling period (dd/mm/yy)
		Soil	Mosses	Algal mats	Dead birds and feathers	
Sôya Coast						
Ongule Islands						
East Ongul Is.	54	52	2	0	2	23/12/2006-01/01/2007
West Ongul Is.	13	8	5	5	0	08/01/2007-09/01/2007<br>22/01/2007-25/01/2007
Ongul Kalven Is.	7	6	1	0	3	10/02/2007
Langhovde	40	14	18	8	5	17/01/2007-21/01/2007
Skarvsnes	125	45	38	51	9	10/01/2007-16/01/2007<br>03/02/2007-09/02/2007
Skallen	11	7	5	2	0	04/01/2007-07/01/2007
Rundvågshetta	14	11	7	1	0	02/01/2007-03/01/2007
Prince Harald Coast						
Riiser-Larsen Peninsula	13	2	10	0	2	19/02/2007
Total	277	145	152	67	21	277

* Some samples consisted of more than one type. Ex.: mosses contained moss thalli and soils.

Table S2. Fungal isolates grown on frozen PDA.

Fungal taxa	Allied species from ITS sequence	Sequence similarity (%)
Ascomycota	*Geomyces* sp. T489/9b (AY345348)	99
	Geomyces sp. C239/10G (AY345347)	99
	Phialocephala sp. 1896 (AM922212)	98
	Phoma herbarum KUC3024 (DQ912692)	100
	Tetracladium sp. CBS 118523 (AJ890435)	96
	Thelebolus sp. BC18 (DQ317351)	99
	Ascomycete sp. K94-020 (EF485234)	99
Basidiomycota	*Dioszegia fristingensis* PYCC 5861^{T} (AY562158)	99
	Mrakia gelida CBS 5272 (KY104296)	99
	Rhodotorula glacialis T8Rg (JQ857032)	99
	Glaciozyma antarctica CBS 5942^{T}	97-100
